# Quality comparison of “Laba” garlic processed by High Hydrostatic Pressure and High Pressure Carbon Dioxide

**DOI:** 10.1038/s41598-020-60667-2

**Published:** 2020-02-28

**Authors:** Dandan Tao, Fangwei Li, Xiaosong Hu, Xiaojun Liao, Yan Zhang

**Affiliations:** 1 College of Food Science and Nutritional Engineering, China Agricultural University, Beijing, 100083 China; 2 National Engineering Research Center for Fruit and Vegetable Processing, Ministry of Science and Technology, Beijing, 100083 China; 3 Department of Food Science and Human Nutrition, University of Illinois at Urbana-Champaign, Urbana, IL 61801 USA

**Keywords:** Biophysical chemistry, Gas chromatography

## Abstract

The production of “Laba” garlic is limited to the homemade method with long processing time and non-uniform color quality. Innovative food processing technologies including high hydrostatic pressure (HHP) and high pressure carbon dioxide (HPCD) were applied to the processing of “Laba” garlic. Products prepared at different treatment pressures (200, 350 and 500 MPa of HHP; 4, 7 and 10 MPa of HPCD) were compared by evaluating the texture, color, flavor and sensory qualities. The results indicated that HHP treatment at 200 MPa was optimal for retaining the textural quality of “Laba” garlic, which was mainly attributed to the compacted cells and the increased Ca^2+^-cross linked cell-cell adhesion. HHP had greater effect on facilitating the formation of the attractive green color of “Laba” garlic than HPCD. The flavor profiles of “Laba” garlic were modified after treatments, with pungent compounds decreased to non-detectable. The results from sensory study confirmed that “Laba” garlic treated by HHP at 200 MPa was most acceptable to consumers. Moreover, considering the treatment capacity and feasibility of commercialization, HHP would be a promising technology in production of “Laba” garlic with improved quality and efficiency.

## Introduction

Garlic (*Allium sativum* L.) has been widely used as food spice and herbal remedy for its edible values and potential medical effect, such as antioxidation, anticancer, and reducing cardiovascular risk^1–4^. “Laba” garlic is one kind of traditional garlic products with unique green color and tasty flavor, which is most favorable in East Asia especially in Northern China^5–7^. Formation of the attractive green color is critical in the processing of “Laba” garlic. It was reported that low-temperature storage of aged garlic and increased permeability of its tonoplast were essential for the greening reaction^6,8–10^. Traditionally, “Laba” garlic was made by immersing garlic cloves in vinegar for weeks during winter time^5^. However, the color of the homemade “Laba” garlic is often light and not uniform. To accelerate the greening of “Laba” garlic, many investigations have been performed. In our previous study, High pressure carbon dioxide (HPCD) has been applied to the processing of “Laba” garlic, which successfully shortened the processing time to 1 d^7^. However, the textural quality of “Laba” garlic was significantly weakened after HPCD treatment, which affected its consumer acceptance. On the other hand, the development of HPCD is on the early stage and most of its studies were conducted in lab scale^11^, the scaling of this innovative technology into production process of “Laba” garlic for the moment would be a problem. Therefore, development of alternative technologies is necessarily needed for the industrial scale production of “Laba” garlic.

High hydrostatic pressure (HHP) has been most extensively studied in the field of non-thermal food processing, and known as one of the most promising non-thermal processes^12–15^. One of the advantages of this technology is that large-scale application of HHP has been commercially available^12^. In addition, its physical damage to cell structure and modification of membrane permeability were reported as main causes of its effect on microorganisms, enzymes and food qualities^16,17^. Previous studies also have reported the effect of HHP on color and flavor changes of garlic products^17–20^. Therefore, HHP is potential to accelerate garlic greening, maintain the texture, and could be an alternative technology for the production of “Laba” garlic.

The goal of this work was to compare the effects of HPCD and HHP processing on textual, flavor, color, and sensory qualities of “Laba” garlic, and to explore an alternative technology in processing the product with improved quality and efficiency.

## Materials and Methods

### Garlic samples preparation

Garlic (*Allium sativum* L.) was harvested in Jinxiang, Shandong province, China. Garlic bulbs were stored at 4~6 °C for 1 month, then were peeled. The cloves in uniform shape and size, free of fungal infection, were selected to be triple-rinsed with distilled water and drained for use.

### HHP processing

HHP processing was achieved by a hydrostatic pressurization equipment (HHP-650, Baotou Kefa Co., Ltd., Inner Mongolia, China). The apparatus contained a pressure vessel of 2.8 liters with a height of 30 cm and an inner diameter of 11 cm. During the operation, distilled water was used as a pressure transfer carrier. A mixture of 100 g of garlic cloves and vinegar (*m*/*v*: 1:1) was vacuum-packed in a sterile polyethylene bag. Then, the bags were placed into the pressure vessel and treated at 200, 350 and 500 MPa for 10 min, respectively. In the pressurization process, the pressure was increased at a rate of 120 MPa/min. In the depressurization process, it was released directly. The pressurization and depressurization time was not counted as HHP processing time (limited to 1 to 3 min). The whole process was completed at room temperature. After the HHP operation, the bags were taken out and immersed in a 55 °C water-bath for 1 hour to accelerate greening. Then, the samples were rapidly cooled and stored at 4~6 °C for further quality analysis.

### HPCD processing

The HPCD equipment was designed by China Agricultural University (Beijing, China). The system consists of a temperature controller, 850 mL pressure vessel, 2TD plunger pump (Hua’an Supercritical Fluid Extraction Co., Ltd., Jiangsu, China) and a pressure gauge. 100 g garlic cloves immersed in vinegar with ratio of 1:1 (*w/v*) were packed in a 250 mL container, and subsequently placed into the pressure vessel. The vessel was pre-heated to 55 °C, and then pressurized by CO_2_ (99.5% of purity, Jing Cheng Co., Beijing, China) with rapid ramp ratio and depressurization ratio (limited to 2 to 3 min). The samples were treated respectively at 4, 7 and 10 MPa for 10 min. After treatment, the mixture of garlic cloves and vinegar were removed out, cooled down and stored at 4~6 °C for 24 h.

### Determination of hardness

A texture analyzer (TA-XT2i, Stable Micro System, UK) was used for measuring hardness. Texture analysis was processed after sample temperature had been stable as room temperature. Samples (10 mm length × 10 mm width × 5 mm height) were cut from the slices and then placed on the base plate. The speed was 1 mm/s during the crosshead pre-test, test, and post-test process and the rest period between cycles was 3 s. The samples were deformed to 30% of the original height. Each set of samples was measured ten times and the average measurement was used as the final result.

### Determination of relative conductivity

Conductivity was determined using the method described in ref. ^10^. with some modifications. Cylindrical samples of 10 mm in diameter were taken from the garlic sample using a stainless steel puncher and they were cut into sheets with a thickness of 1 mm for measurement. The samples were rinsed with ddH_2_O and then wiped up. Garlic sheets (2.5 g) were placed in a test tube with 30 mL of ddH_2_O in it and then put in the shaking incubator at 25 °C for 30 min. A conductivity meter (EC215, Hanna, Italy) was used for testing the electrical conductivity. The tubes were placed in boiling water for 10 minutes to destroy the tissue. After cooling the sample to room temperature, the total electrolyte content of the tissue (expressed as total conductivity) was measured. The relative conductivity of the samples was expressed as the ratio of conductivity before and after the destruction.

### Immunolabelling of pectic epitopes

Immunolabelling was conducted using the method described in ref. ^21^. with some modifications. Garlic samples were cut into 100~300 μm thickness sections and placed in 70% (v/v) ethanol for 2 h. The immunolabelling of pectic epitopes in these cryosections started with an incubation with primary antibody (JIM5, LM19, LM20 and 2F4, tenfold diluted in 3% MPBS) for 1.5 h at room temperature. Then secondary labelling with anti-rat Ig antibody coupled to fluorescein isothiocyanate (FITC) (Sigma, St. Louis, Mo., USA) was diluted 1/20 in 3% MPBS and used for the visualization of JIM5, LM19 and LM20. Labelling with 2F4 was made visible with an anti-mouse IgG antibody coupled to FITC (Sigma, St. Louis, Mo., USA) diluted 1/50 in 3% MPBS. After primary labelling, sections were washed with PBS for 15 min, transferred to second labelling with an incubation at room temperature for 1 h. After a final washing step with PBS, sections were mounted on glass slides and examined with a Zeiss LSM780 confocal microscope (Zeiss, Oberkochen, Germany). Two different garlic samples were examined for each treatment group.

### Analysis of volatile compounds using GC-MS

The extraction and concentration of volatile compounds in garlic was realized by headspace solid phase microextraction (SPME). The SPME procedure was refered to the method described in ref. ^19^. with some modification. Garlic samples (7 g each) were fully crushed and placed in a 50 mL glass vial containing a magnetic stirrer and 7 mL ultra-pure water. Then the vials were sealed with screw tops and put onto the heating platform at 30 °C for 30 min for equilibration. A Carboxen Polydimethylsiloxane (CAR/PDMS) SPME fiber head used for volatile extraction was inserted into the vial and exposed to the headspace. After 15 min of sampling at 30 °C, the head was retracted and immediately inserted into the inlet of sample-injection hole for desorption, and desorption was conducted at 250 °C for 5 min in split mode (20:1).

Chromatographic separations were performed on GC-MS instrument (7890-A-5975C, Agilent Technologies, Inc., USA), which was equipped with a WAX column (30 m × 0.25 mm i.d. × 0.25 μm; J&W Scientific, Folsom, CA, USA). The flow rate of high-purity helium (purity >99.999%) as carrier gas was 1.0 mL/min. The oven temperature was set from 40 °C for 4 min, increasing 6 °C/min to 220 °C, and then maintained for 3 min. The mass detector operated in electronic impact (EI) mode of 70 eV and the temperatures for the source and interface were 230 °C and 280 °C, respectively; the consecutive scanning scope was over a mass range of m/z 33–450 Amu. The volatile compounds were identified by comparing their mass spectra with the MS library of NIST 08 (match quality >80) and with those of reference compounds and published data. The compositions were further identified with the mass spectra library combined with the retention time, mass spectrum and actual compositions. The qualitative result was estimated as the relative percentage of given compounds based on integrated ion areas divided by the chromatograms total ion area.

### Electronic nose measurements

An Electronic Nose instrument (Heracles, Alpha MOS, Toulouse, France) combined with a headspace auto-sampler was used for the analysis of samples. Prior to testing, the electronic nose system was preheated and calibrated with pure air until the data received by the sensor was stable. Garlic samples (3 g each) were crushed and placed into 10 mL glass vial with silicone caps and settled into the automatic sampling carousel. The measurement was started after equilibration at 65 °C for 10 min under stirring (350 rpm). The sample headspace was pumped over the sensor surfaces for 120 s. After analysis, the system was purged with pure air prior to the next injection. The maximum response points recorded were automatically used as the electronic nose response. Each measurement was executed for three times and the average result was used for data analysis^22^.

### Determination of alliinase activity

Alliinase activity was measured based on the method described in ref. ^7^. 25 g of the garlic cloves were mixed with 35 mL of pre-cooled Hepes buffer (0.05 M, pH 6.9). The samples were then crushed for 2 minutes, filtered, and centrifuged at 10,000×rpm for 10 minutes at 4 °C. The supernatant was fetched and measured. The alliinase activity was showed by pyruvate formation. The details were executed according to the method of ref. ^7^.

### Color measurements

50 g of the treated garlic samples were accurately weighed and mixed with 50 mL of distilled water. The mixture was mashed for 3 minutes with a stirrer (JYDZ-31B, Jiuyang Co. Ltd China). The CIE L^*^a^*^b^*^ color evaluation was measured by a Color Difference Meter (ColorQuest XE, HunterLab, USA) from the reflectance spectra. The mashed sample was placed in an optical glass tray and the next measurement was performed according to the method of Tao^7^. The sample was vacuum filtered through a double filter paper and then filtered through a 0.45 μm membrane. The filtrate was placed in a quartz cuvette for absorbance measurement using a UV spectrophotometer UV-1800 (Shimadzu Co., Tokyo, Japan) at 590 nm^5^.

### Sensory evaluation

The sensory testers were 12 students from China Agricultural University who were trained in sensory evaluation. 20 g of complete “Laba” garlic cloves from each group were put into 50 mL plastic cups. The cup was marked by three random digits. The testers were asked to rate for specific sensory aspects i.e. color, flavor, and texture using a 9-point scoring scale based on the method described in ref. ^7^.

### Statistical analysis

Data analysis were performed by IBM SPSS Statistics 20.0 (IBM, Armonk, NY, USA) and plotted using Microcal Orign software 8.6 (Microcal Software, Inc., Northampton, MA, USA), ANOVA tests were performed to determine the significance at 95% confidence. Each experiment was carried out in triplicates.

## Results and Discussion

### Texture quality of “Laba” garlic treated by HHP and HPCD

The texture of garlic can directly be expressed by hardness. The change of relative conductivity will help to elucidate the effect of high pressure on texture. As shown in Table 1, relative conductivity significantly increased with the increasing of treatment pressure, indicating the increase of the permeability of garlic cell membrane^10^. Specifically, it increased to 2.37 and 2.48 times of the untreated samples after HPCD treatment at 7 and 10 MPa, which were significantly higher than those treated by HHP at 200 MPa (1.69 times). It was assumed that the low pH and the existence of CO_2_ in HPCD were accounted for its greater effect on modification of cell membrane permeability and cell wall destruction. The CO_2_ in this research was in a supercritical state with high osmotic and solvating effects^23^. Therefore, it also had a strong penetrating ability to the cell membrane and cell wall, which was beneficial to accelerating the green reaction of garlic.

**Table 1:** Effects of HHP and HPCD on texture properties, conductivity and alliinase activity of “Laba” garlic.

Indices	Untreated	HHP			HPCD		
		200 MPa	350 MPa	500 MPa	4 MPa	7 MPa	10 MPa
Hardness (Kg)	10.13 ± 0.42^a^	7.94 ± 0.59^c^	5.39 ± 0.40^e^	6.93 ± 0.32^d^	8.94 ± 0.24^b^	7.71 ± 0.36^c^	6.59 ± 0.45^d^
Relative conductivity (%)	35.48 ± 3.48^d^	57.92 ± 1.48^c^	67.02 ± 3.05^b^	79.24±4.45^a^	59.03 ± 2.39^c^	84.03 ± 4.30^a^	88.02 ± 3.49^a^
Alliinase activity (U/mg)	101.21 ± 2.94^a^	85.32 ± 1.29^b^	75.19 ± 1.38^c^	48.33 ± 1.54^f^	62.62 ± 1.58^d^	53.43 ± 2.04^e^	32.31 ± 1.74^g^

**Figure 1:**
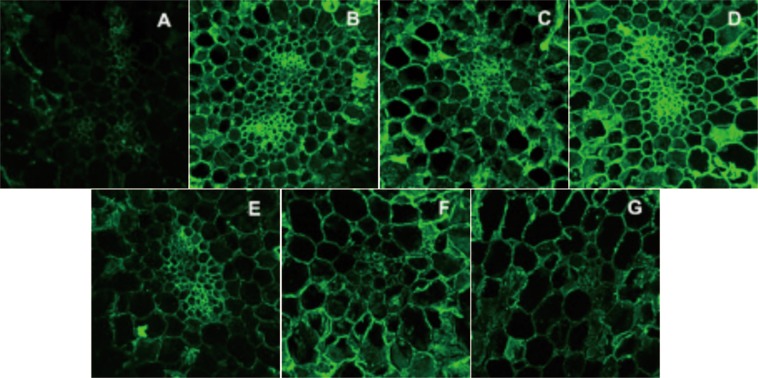
*In situ* immunofluorescence labelling of high pressure processed garlic cell with LM 19 (**A**- untreated; **B**- HHP treatment at 200 MPa; **C**- HHP treatment at 350 MPa; **D**- HHP treatment at 500 MPa; **E**- HPCD treatment at 4 MPa; **F**- HPCD treatment at 7 MPa; **G**- HPCD treatment at 10 MPa).

**Figure 2:**
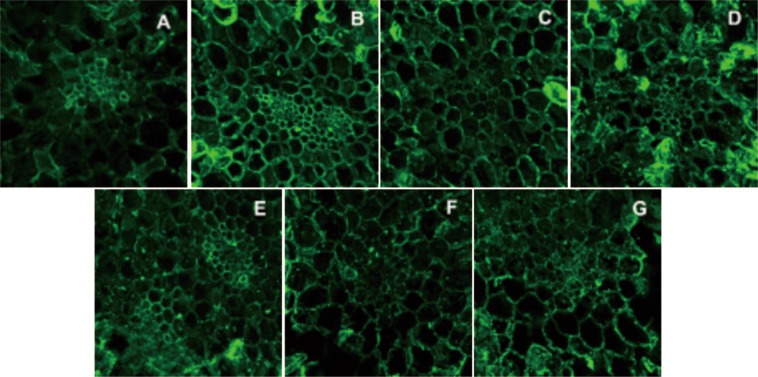
*In situ* immunofluorescence labelling of high pressure processed garlic cell with 2F4 (**A**- untreated; **B**- HHP treatment at 200 MPa; **C**- HHP treatment at 350 MPa; **D**- HHP treatment at 500 MPa; **E**- HPCD treatment at 4 MPa; **F**- HPCD treatment at 7 MPa; **G**- HPCD treatment at 10 MPa).

Due to the increase of cell membrane permeability and cell disruption induced by HHP and HPCD, conversions of pectin may be facilitated, which will contribute to the changes in the cell wall structure, affecting the textural quality of garlic products. Defined monoclonal antibodies against the pectins are useful for both morphologic analysis and pectin distribution analysis^24^. LM20, JIM5, LM19 and 2F4 were used to investigate the distribution of pectin in the cell wall of garlic. In this section, the most apparent observations (LM19 and 2F4) were discussed, while labelling patterns of LM20 and JIM5 were not included in discussion. Labelling of samples with the two anti-pectin antibodies (LM19 and 2F4) are illustrated in Figs. 1 and 2. The labelling of samples with LM19 represents one of the most abundant pectin of cell walls. According to Fig. 1, significant changes of cell morphological behavior were observed. In comparison, the most compressed cells were observed in samples treated by HHP at 200 MPa, while the most expanded cells were seen in the HPCD group at 10 MPa. It can be clearly seen that the whole cell wall, the region of the cell wall lining, and the intercellular spaces were labeled with LM19 in HHP-treated at 200, 350 and 500 MPa. And faint labelling of the corners of tricellular junctions as well as some parts of the middle lamella were only noticed in the untreated samples, which was different from the reports of asparagus lettuce^25^. Localization of Ca^2+^-cross-linked pectin was visualized by labelling of samples with 2F4. As shown in Fig. 2, HPP and HPCD both resulted in a pronounced change in the labelling pattern of 2F4 compared to the untreated. After treatments, 2F4-epitopes not only occurred in the cell junctions but also in other parts of the cell wall. In addition, the border of cell-cell cohesion was most clear in labelling of the samples treated by HHP at 200 MPa. It was concluded that HHP had less effect on inducing morphological changes in garlic than HPCD, and greater effect on increasing the Ca^2+^ -cross linked cell-cell adhesion. The results were in consistence to the reports from refs. ^21,25^.

Researchers simulated the traditional process of “Laba” garlic by soaking garlic cloves in acetic acid solution, and found that the acetic acid increased the permeability of cell membrane^10^. In this investigation, the tissue damage induced by high pressure helped to form the textural quality of “Laba” garlic. However, excessive tissue damage induced the significant loss of hardness, which would negatively affect consumer acceptance^7^. Cell damage, redistribution of pectin both contributed to the modifications in textural quality of “Laba” garlic. However, the results of HHP and HPCD treatments are quite different. The effect of HHP on compacting cells and increasing the cell-cell adhesion were useful for protecting the final texture from softening, while the effect of HPCD on expanding cells and destroying cell integrity might have weakened the texture. In comparison, HHP was superior to HPCD for the retention of the textural quality because of its better protection of cell structure. Among all HHP treatments, the greatest increase of Ca^2+^ -cross linked cell-cell adhesion induced by 200 MPa. As a result, HHP treatment at 200 MPa was most favorable for the formation of texture quality of “Laba” garlic.

### Flavor profile of “Laba” garlic treated by HHP and HPCD

A total of 14 odor-active compounds were detected in untreated garlic. The relative contents of each violative compound are shown in Table 2. Among all these typical volatile compounds detected in the untreated samples, content of diallyl disulfide (DADS, 53.70% of the relative peak area) was the highest, followed by 1-oxa-4,6-diazacyclooctane (26.80%), allyl methyl disulphide (9.27%), 1,3-dithiane (1.32%), 3-vinyl-1,2-dithiocyclohex-4-ene (0.71%), diallyl sulfide (0.63%), 3,4-dimethylthiophene (0.61%), allyl mercaptan (0.26%), 1,1-allyl sulfide (0.18%), dimethyl disulfide (0.14%), 2,5-dimethylthiophene (0.12%), allyl methyl sulfide (0.06%), dimethyl trisulfide (0.06%) and 3-vinyl-1,2-dithiocyclohex-5-ene (0.05%). The total relative peak area of main sulfides was 63.86%. Despite main sulfides, trisulfide, di-2-propenyl (PeTS) and diallyl tetrasulphide (DATTS) were also repoted as critical compounds of garlic because of their low threshold values^19^. However, these compounds were not detected in this study. The total relative content of main sulfides decreased for samples treated by HHP and HPCD. With the increase of treatment pressure, the contents of allyl methyl disulphide, allyl methyl sulfide, dimethyl disulfide and dimethyl trisulfide decreased to non-detectable level. As the relative content of main volatile compounds was positively correlated to the odor intensity, it was indicated that the intensity of the characteristic pungent odor of garlic decreased with treatment pressure^19^. In this case, the flavor profile of “Laba” garlic could be modified by the pressure intensity of HHP or HPCD, which is important for achieving the consumer acceptance. It was reported that the main sulfides were produced from degradation of allicin, which was resulted from enzymatic reactions involving alliinase^26^. In intact garlic cells, alliinase and alliin were located in the vacuole and cytoplasm, respectively. Only when cell damage happens, the alliinase can contact with its substance alliin and induce enzymatic reactions. The changes of alliinase activity after HHP and HPCD treatments are shown in Table 1. It can be seen that alliinase activity was inactivated rapidly with the increasing of pressure, in accordance with the changes in total relative content of main sulfides. HPCD treatments (4, 7 or 10 MPa) had greater effect on inactivating the enzyme than HHP treatments (200 or 350 MPa), and the lowest inactivation of alliinase was from HHP treatment at 200 MPa (15.70%). The samples treated by HPCD at 10 MPa had the lowest total relative content of main sulfides and highest inactivation of alliinase. It can be inferred that the reduction in the relative content of main sulfides after HHP and HPCD treatments were attributed to the inactivation of alliinase, and less production of allicin^27,28^.

**Table 2:** Relative contents of main volatile compounds in“Laba” garlic processed by HHP and HPCD.

Compound	Relative content (Area %)						
	Untreated	HHP 200 MPa	HHP 350 MPa	HHP 500 MPa	HPCD 4 MPa	HPCD 7 MPa	HPCD 10 MPa
Allyl mercaptan	0.26 ± 0.02	0.12 ± 0.02	0.30 ± 0.00	0.24 ± 0.02	0.19 ± 0.02	0.23 ± 0.03	0.22 ± 0.03
Allyl methyl sulfide	0.06 ± 0.00	0.09 ± 0.01	—	—	0.06 ± 0.00	0.07 ± 0.00	—
Dimethyl disulfide	0.14 ± 0.01	0.07 ± 0.00	—	—	0.03 ± 0.00	—	—
Diallyl sulfide	0.63 ± 0.02	0.31 ± 0.01	0.23 ± 0.03	0.14 ± 0.04	0.63 ± 0.01	0.23 ± 0.03	0.21 ± 0.04
1,1-Allyl sulfide	0.18 ± 0.01	0.38±0.00	0.59 ± 0.05	0.42 ± 0.09	0.34 ± 0.02	1.07±0.06	1.41 ± 0.01
3,4- Dimethylthiophene	0.61 ± 0.01	0.84±0.10	1.21 ± 0.11	1.19 ± 0.07	0.63 ± 0.05	0.15 ± 0.01	0.14 ± 0.04
2,5- Dimethylthiophene	0.12 ± 0.02	0.16 ± 0.00	0.42 ± 0.02	0.38 ± 0.04	0.47 ± 0.00	0.30 ± 0.01	0.27 ± 0.02
Allyl methyl disulphide	9.27 ± 0.02	5.12 ± 0.02	—	—	4.82 ± 0.27	—	—
1,3- Dithiane	1.32 ± 0.04	1.26 ± 0.01	1.19 ± 0.02	2.52 ± 0.03	0.95 ± 0.04	1.23 ± 0.09	1.41 ± 0.05
Diallyl disulfide	53.70 ± 1.77	55.47 ± 0.84	24.40 ± 1.14	20.86 ± 1.07	43.62 ± 1.42	23.31 ± 1.08	21.15 ± 1.10
1-Oxa-4,6-diazacyclooctane-5-thione	26.80 ± 0.03	27.42 ± 0.32	28.79 ± 1.01	33.54 ± 1.47	29.30 ± 0.83	24.03 ± 1.16	24.63 ± 1.25
3-Vinyl-1,2-dithiocyclohex-5-ene	0.05 ± 0.00	0.29 ± 0.18	0.36 ± 0.03	0.14 ± 0.01	0.14 ± 0.02	0.21 ± 0.04	0.22 ± 0.00
3-Vinyl-1,2-dithiocyclohex-4-ene	0.71 ± 0.00	2.07 ± 0.01	3.83 ± 0.02	1.95 ± 0.11	1.87 ± 0.01	4.17 ± 0.01	3.85 ± 0.08
Dimethyl trisulfide	0.06 ± 0.01	0.07 ± 0.00	0.08 ± 0.00	—	0.05 ± 0.00	0.25 ± 0.11	0.26 ± 0.04
Total amount of sulfides	93.91 ± 1.77	93.67 ± 0.92	61.40 ± 1.53	61.38 ± 1.83	83.10 ± 1.67	55.25 ± 1.59	53.77 ± 1.67

Note: Results were presented as the mean ± standard deviation (n = 3); —, not detected.

**Figure 3:**
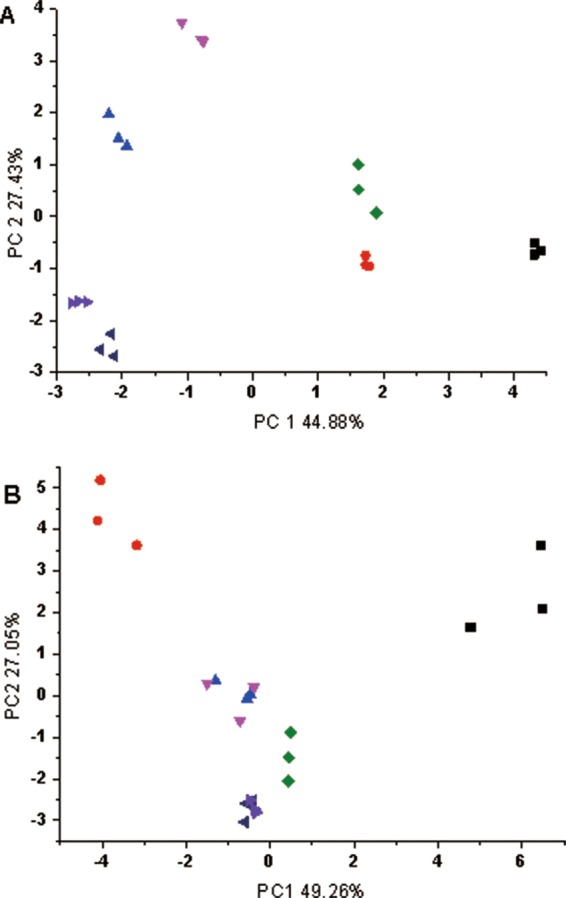
PCA plot of volatile compounds in “Laba” garlic based on data from A- GC-MS; and B- electronic nose (- CK; - HHP treatment at 200 MPa; - HHP treatment at 350 MPa; - HHP treatment at 500 MPa; - HPCD treatment at 4 MPa; - HPCD treatment at 7 MPa; - HPCD treatment at 10 MPa).

Principal component analysis (PCA) is a statistical technique capturing the relevant information in a set of data in a lower dimension. The PCA plots of volatile compounds in garlic after HHP and HPCD treatments are shown in Fig. 3. Figure 3A presented the results from GC-MS analysis, with the primary two components PC1 being 44.88% and PC2 27.43%, respectively. It can be seen that all datasets were clearly separated, indicating that there was obvious difference in the volatile compounds between the samples treated with HHP, HPCD and the untreated. Figure 3B are results from electronic nose analysis, in which PC1 accounted for 49.26% of the variance in the data-set and PC2 accounted for 27.05%. Five clusters were clearly separated, in which HPCD-treated samples at 7 and 10 MPa were in the same cluster and HHP-treated samples at 350 and 500 MPa were in the same cluster.

### Color quality of “Laba” garlic treated by HHP and HPCD

The color indices of “Laba” garlic treated by HPCD and HHP are shown in Table 3. Among CIE L^*^a^*^b^*^ values and hue angle (*H*^0^), a^*^ is a representing index for red (+) and green color (−). For “Laba” garlic, the smaller in a^*^ value (−) the darker in green color^7^. The initial a^*^ was 1.52 ± 0.08 (data not shown). It can be seen that a^*^ value decreased significantly after HPCD and HHP treatments. Among HPCD treated samples, a^*^ value of samples treated at 7 and 10 MPa were significantly lower than that treated at 4 MPa, but significantly higher than samples treated by HHP at 200, 350 or 500 MPa (*p* < 0.05). It was indicated that HHP treatments combined with subsequent treatment (heated at 55 °C for 1 h) had greater effect on facilitating garlic greening than HPCD treatments. Aside from a^*^ value, *H*^0^ and A_590 nm_ are indices for evaluating greening of garlic^18^. However, no significant differences were found in the results of *H*^0^ and A_590 nm_ among samples treated by HPCD and HHP.

**Table 3:** Effects of HHP and HPCD on color indices of “Laba” garlic.

Indices	HHP			HPCD		
	200 MPa	350 MPa	500 MPa	4 MPa	7 MPa	10 MPa
L^*^	32.15 ± 0.55^b^	32.14 ± 1.13^b^	31.95 ± 0.74^b^	35.78 ± 1.03^a^	34.27 ± 0.86^a^	31.16 ± 0.48^b^
a^*^	−8.16 ± 0.61^c^	−8.23 ± 0.49^c^	−7.89 ± 0.73^c^	−3.22 ± 0.92^a^	−6.14 ± 0.69^b^	−6.39 ± 0.42^b^
b^*^	0.59 ± 0.14^c^	0.61 ± 0.10^c^	0.53 ± 0.11^c^	1.13 ± 0.18^a^	0.85 ± 0.23^b^	0.89 ± 0.21^b^
*H*^0^	175.88 ± 1.18^a^	173.10 ± 0.89^a^	176.17 ± 1.31^a^	135.01 ± 1.73^b^	172.14 ± 1.49^a^	172.09 ± 1.92^a^
A_590 nm_	2.13 ± 0.39^a^	2.09 ± 0.31^a^	1.95 ± 0.28^a^	1.54 ± 0.21^b^	1.93 ± 0.38^a^	1.98 ± 0.17^a^

Note: Results were presented as the mean ± standard deviation (n = 3); different lowercase letters in the same row represents significant difference (p < 0.05); the measurements were made in triplicate.

### Sensory quality of “Laba” garlic treated by HHP and HPCD

**Figure 4:**
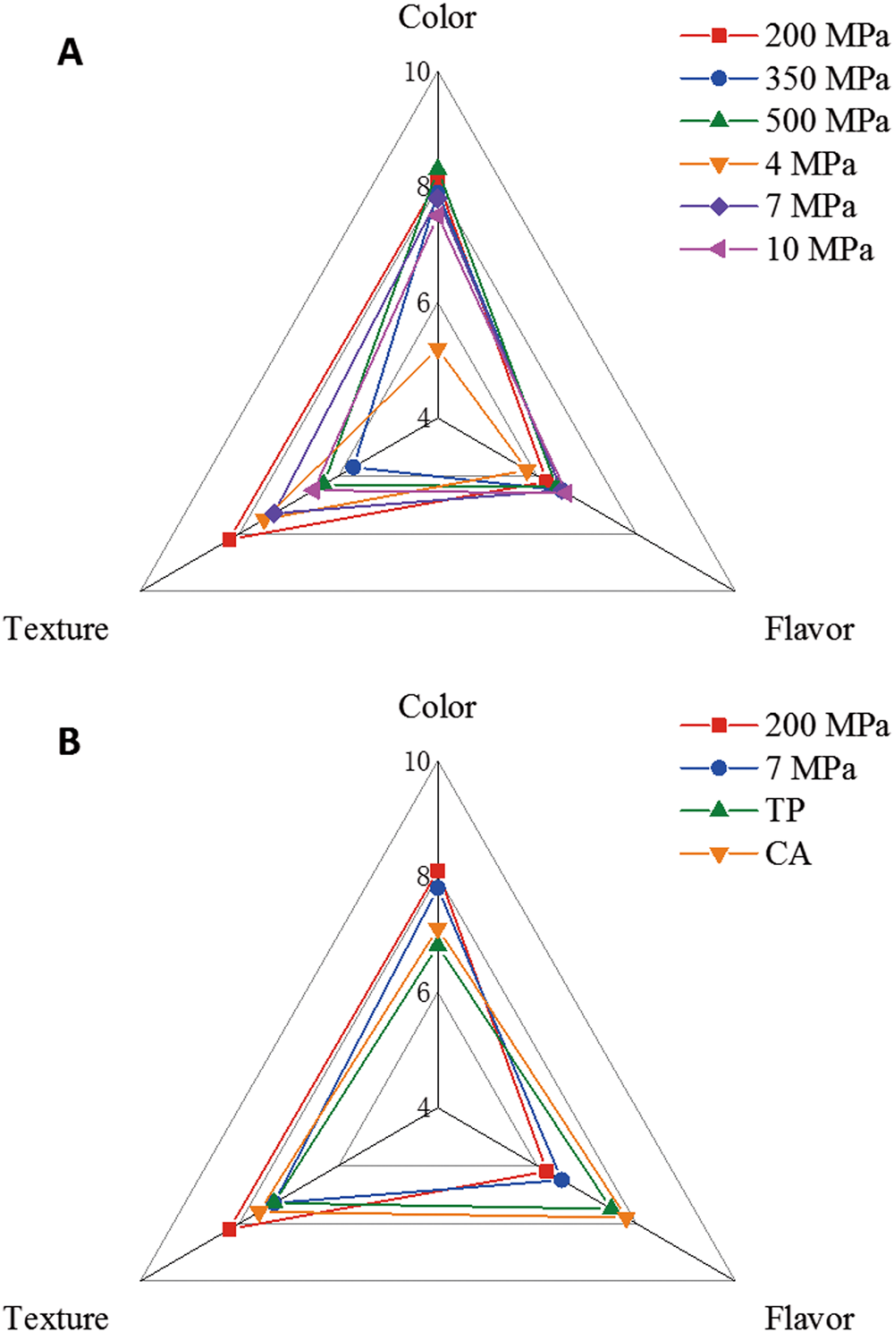
Spider plot for sensory profiles of “Laba” garlic based on data from (**A**)- HHP and HPCD treatments; and (**B**)- traditional treatments (TP- Traditional processing; CA- Commercially available).

Sensory results of “Laba” garlic treated by different methods are shown in Fig. 4. Three indices i.e. color, flavor and texture were included in the sensory test. Results of color, flavor and texture scores for HHP-treated and HPCD-treated “Laba” garlic are shown in Fig. 4A. No significant differences between samples treated at 200, 350 and 500 MPa were found in results of color and flavor scores (*p*> 0.05). However, a significant higher score on texture score was achieved at 200 MPa in HHP group (*p* < 0.05). On the other hand, For the HPCD-treated “Laba” garlic, it can be seen that the color and flavor scores of samples treated at 4 MPa were lower than that of samples treated at 7 and 10 MPa, while the texture score of samples treated at 10 MPa were lower than that of samples treated at 4 and 7 MPa. The highest sensory score of “Laba” garlic treated with HPCD method achieved at 7 MPa. In comparison, the overall sensory quality of “Laba” garlic treated at 200 MPa by HHP was superior to that treated at 7 MPa by HPCD because of the higher score on textural quality. The sensory comparison of “Laba” garlic among by high-pressure processing (200 MPa of HHP treatment; 7 MPa of HPCD treatment), traditional processing and commercial available are shown in Fig. 4B. It can be seen that the “Laba” garlic processed by HPCD or HHP had higher scores in color and texture, with highest scores obtained from samples treated by HHP at 200 MPa. In terms of flavor, the highest score was from commercially available products, followed by traditional processing garlic, which might be attributed to richer flavor profiles formed during longer processing time than HHP and HPCD treatments. Based on the results of total relative content of total sulfides as shown in Table 2, the total relative content of main sulfides at 200 MPa and 4 MPa were significantly higher than the other groups treated by HHP and HPCD respectively, making the flavor more irritating. Among all the high-pressure processing groups, the highest sensory score was obtained from samples treated by HHP at 200 MPa. However, the flavor quality of “Laba” garlic treated by HHP needs to be improved in compared with those processed from traditional methods with longer time.

### Discussion

The main finding in this work is that HHP treatment, in particular at 200 MPa, has greater effect on retaining the color and texture quality of “Laba” garlic than HPCD treatment. However, there are a few limitations need to be considered. The current experiment was designed for practical purposes. The developed methods combining high pressure (HHP and HPCD) and vinegar, though are helpful for reducing the procedures and potential risks of cross-contamination in processing of the product, made it difficult to analyze the effects of individual parameter (e.g. pressure) on the changes of product quality. Also, the effects of come-up-time (CUT) in HHP and HPCD treatments for each pressure studied are not clear. Therefore, more researches need to be done in future to understand how parameters (e.g. pressure, temperature, time) and CUT in HHP and HPCD treatments influence the quality of “Laba” garlic. In addition, improving the flavor profile of “Laba” garlic treated by HHP to better meet consumer acceptance would also be a critical topic to explore in future study.

## Conclusions

HHP shortened the processing time of “Laba” garlic from weeks to one day by accelerating the formation of green pigments. The highest sensory scores in color and texture were achieved at 200 MPa of HHP. Particularly, HHP had better capability to retain its textural quality than HPCD, which was mainly attributed to the compacted cell structure and the increase of cell-cell inhesion induced by HHP. The relative content of main sulfides decreased with the increasing of treatment pressure, indicating the flavor of “Laba” garlic was differently modified by HHP with different pressures. The changes in garlic flavor compounds were favorable to the quality formation of “Laba” garlic according to the sensory results. The overall study on color, textural attributes and sensory quality of garlic indicated that HHP could be a more promising innovative technology in processing “Laba” garlic than HPCD. Moreover, it is more feasible for commercial production considering that the HHP was more scalable than HPCD in industry applications.
